# 3’Pool-seq: an optimized cost-efficient and scalable method of whole-transcriptome gene expression profiling

**DOI:** 10.1186/s12864-020-6478-3

**Published:** 2020-01-20

**Authors:** Gabriel Sholder, Thomas A. Lanz, Robert Moccia, Jie Quan, Estel Aparicio-Prat, Robert Stanton, Hualin S. Xi

**Affiliations:** Computational Sciences, Medicinal Sciences, Pfizer, Inc., Cambridge, MA 02139 USA

**Keywords:** Next generation sequencing, RNA-seq, Transcriptomics, 3′-RNA sequencing, 3’Pool-seq, Differential gene expression

## Abstract

**Background:**

The advent of Next Generation Sequencing has allowed transcriptomes to be profiled with unprecedented accuracy, but the high costs of full-length mRNA sequencing have posed a limit on the accessibility and scalability of the technology. To address this, we developed 3’Pool-seq: a simple, cost-effective, and scalable RNA-seq method that focuses sequencing to the 3′-end of mRNA. We drew from aspects of SMART-seq, Drop-seq, and TruSeq to implement an easy workflow, and optimized parameters such as input RNA concentrations, tagmentation conditions, and read depth specifically for bulk-RNA.

**Results:**

Thorough optimization resulted in a protocol that takes less than 12 h to perform, does not require custom sequencing primers or instrumentation, and cuts over 90% of the costs associated with TruSeq, while still achieving accurate gene expression quantification (Pearson’s correlation coefficient with ERCC theoretical concentration r = 0.96) and differential gene detection (ROC analysis of 3’Pool-seq compared to TruSeq AUC = 0.921). The 3’Pool-seq dual indexing scheme was further adapted for a 96-well plate format, and ERCC spike-ins were used to correct for potential row or column pooling effects. Transcriptional profiling of troglitazone and pioglitazone treatments at multiple doses and time points in HepG2 cells was then used to show how 3’Pool-seq could distinguish the two molecules based on their molecular signatures.

**Conclusions:**

3’Pool-seq can accurately detect gene expression at a level that is on par with TruSeq, at one tenth of the total cost. Furthermore, its unprecedented TruSeq/Nextera hybrid indexing scheme and streamlined workflow can be applied in several different formats, including 96-well plates, which allows users to thoroughly evaluate biological systems under several conditions and timepoints. Care must be taken regarding experimental design and plate layout such that potential pooling effects can be accounted for and corrected. Lastly, further studies using multiple sets of ERCC spike-ins may be used to simulate differential gene expression in a system with known ground-state values.

## Background

Transcriptional profiling by RNA sequencing (RNA-seq) has proved to be a powerful tool for examining the effects of genetic and chemical perturbations on biological systems [1–5]. Typically, RNA-seq is carried out by purifying RNA and subjecting it to one of many commercial Next Generation Sequencing (NGS) preparation kits [6–8]. These kits create libraries that consist of fragmented cDNA with an average length of 300–500 bases, where each fragment is flanked with indexed adapters that are required for flow-cell binding inside the sequencer and subsequent sample demultiplexing. One of the most widely used kits for sequencing mRNA is TruSeq [6–8], which uses salt-catalyzed hydrolysis, random priming, and end repair/ligation to create sequence-ready libraries from bulk RNA [9]. Another is SMART-seq [10], which utilizes the template-switching activity of reverse transcriptase in conjunction with anchored oligo-dT primers to create and amplify full-length cDNA from as few as one cell. This product is subsequently fragmented and tagged with adapters in a transposase-mediated process called tagmentation [10, 11] to complete the library preparation process.

While the mechanistic details of these two methods differ, they both share the attribute of yielding NGS-compatible libraries that give full-length transcript data. Given the average mammalian transcript length of approximately 2700 bases [12], most transcripts will yield around six fragments that are all sequenced in parallel. As a result, full-length sequencing is able to give information about splice variants and sequence diversity [13, 14], although it yields redundant data if one’s main goal is to determine differential expression at the gene level.

Because the costs of full-length library preparation and sequencing often exceed $160 per sample, financial considerations are often limiting determinants regarding experiment design. As such, several groups have committed substantial resources towards developing more affordable, alternative RNA-seq library preparation methods. One alternative method of note is 3′-end sequencing [15], which preferentially amplifies and sequences only the 3′-end of RNA transcripts. Because each transcript contributes only one fragment for sequencing, approximately 5–6 times as many samples can be combined per sequencing run and yield the same relative read depth per gene as compared to full-length sequencing. While commercial 3’RNA-seq kits exist (for example, QuantSeq from Lexogen, Inc.) and do reduce sequencing costs, the protocols lack an early pooling step that decreases sample number and the preparation costs still exceed $25 per sample, making them unsuitable for large studies.

The utility of 3′ sequencing is clearly demonstrated by Drop-seq [16], a single-cell RNA-seq method that utilizes SMART-seq technology, bead-conjugated primers, and microfluidics to allow the user to amplify 3′-end fragments and maintain single-cell identity from over 30,000 cells at once. Although Drop-seq and its related microfluidics-based workflows are at the forefront of single-cell sequencing technology [17], their protocols have not been optimized for preparing libraries from bulk RNA in standard tube or plate format. Furthermore, the requirement of custom primers during sequencing makes them unfeasible for researchers who use NGS services that prohibit the use of non-standard sequencing primers, or who wish to share a sequencing run with other types of libraries. Recent studies have attempted to utilize 3’RNA-seq technology for plate-based transcriptomics profiling of bulk RNA [18, 19], but they require custom sequencing reagents and expensive instrumentation, and thorough benchmarking against standard RNA-seq protocols is either lacking or is shown to be suboptimal (see discussion).

Herein, benchmark RNA from wild-type and GFAP-IL6 mice along with ERCC RNA standards were utilized to design and optimize a process called 3’Pool-seq, which draws from aspects of SMART-seq, Drop-seq, and TruSeq, and does not require custom sequencing primers or instrumentation. 3’Pool-seq allows the user to create and sequence 3′-mRNA libraries in under a day for less than $15 per sample ($3 library preparation and $12 sequencing cost per sample), while still maintaining a standard of quality with regard to data generation and gene expression quantification that is on par with TruSeq. The robustness of 3’Pool-seq was further demonstrated with as little as 10 ng input RNA. This method was then applied in a plate-based fashion to profile the transcriptomic changes that occur when HepG2 cells are treated with PPARγ agonist drugs, and successfully distinguished troglitazone from pioglitazone by its unique transcriptomic signature corresponding to cytotoxicity.

## Results

### Design of 3’Pool-seq

**Fig. 1:**
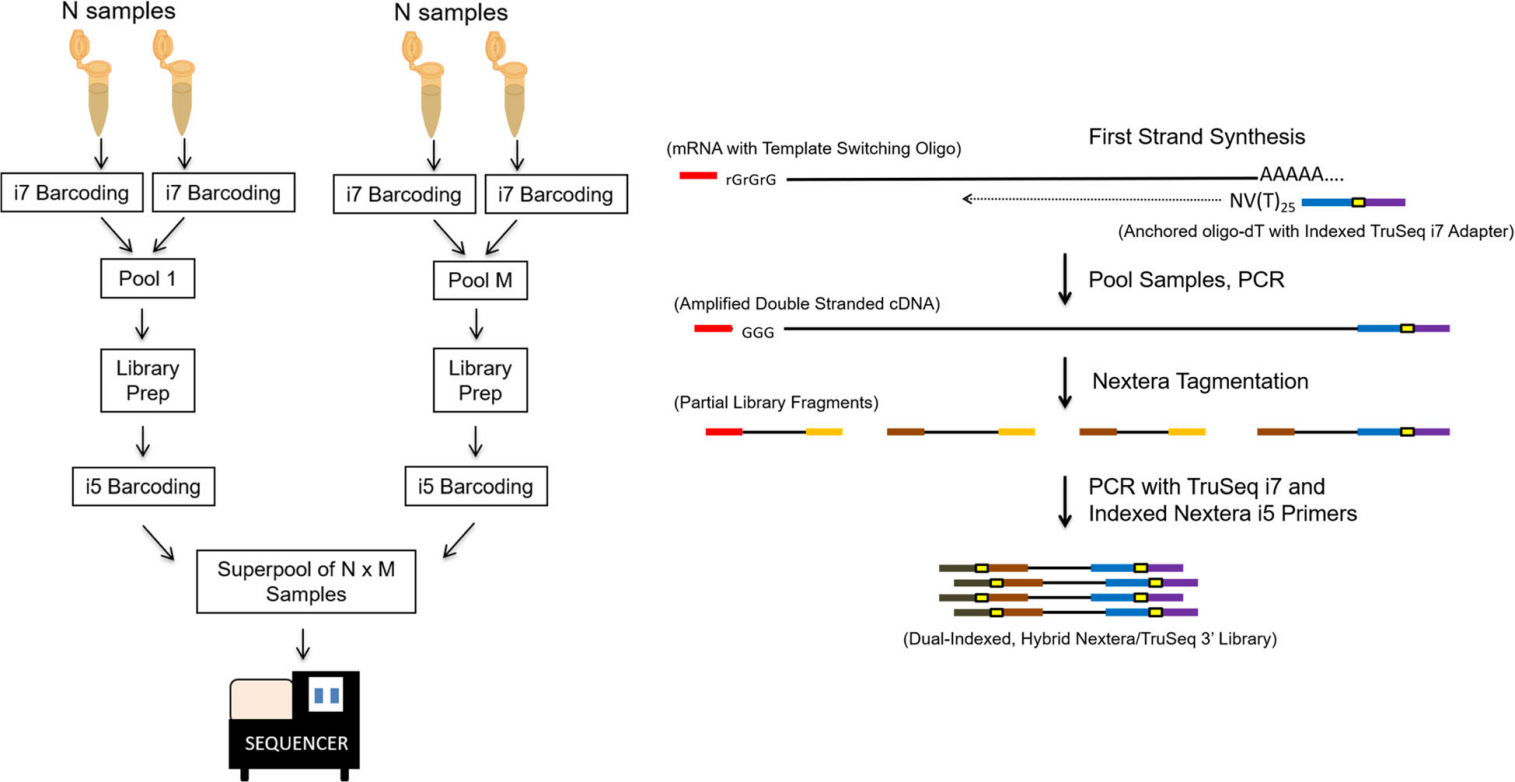
A schematic representation of the 3’Pool-seq protocol. The use of anchored oligo-dT primers with standard indexed TruSeq i7 adapter overhangs for first strand synthesis allows immediate pooling of multiple samples after reverse transcription. Within a pool, each sample can be uniquely identified by the TruSeq i7 index. Once pooled, purification, PCR, and Nextera tagmentation reagents are used to generate cDNA fragments. A second PCR step using standard TruSeq i7 and indexed Nextera i5 adapters allows selective amplification of only 3′-end cDNA fragments and barcoding of each sample pool with a standard Nextera i5 index. The final product is a dual-indexed hybrid Nextera/TruSeq 3′-library where the i5 Nextera index serves as the pool index, and the i7 TruSeq index serves as the sample index within a pool. Multiple indexed library pools can be further quantified and combined in equal proportions into a superpool for sequencing

A schematic representation of the 3’Pool-seq method for gene expression quantification is depicted in Fig. 1. Total RNA from each input sample is first reverse-transcribed into cDNA using an anchored oligo-dT primer with an indexed TruSeq i7 adapter overhang. These indices serve as 3′-end barcodes for the individual samples. The same Template Switching Oligo that is used in SMART-seq is added to the reaction to provide a handle at the 3′-end of the cDNA to allow full-length cDNA amplification. However, in contrast to the standard SMART-seq protocol, cDNA samples with unique 3′-end barcodes are pooled immediately after the first strand cDNA synthesis. Subsequent library preparation steps (cDNA amplification, Nextera tagmentation, 3′-end cDNA fragment amplification) are then carried out on the sample pools, drastically reducing the time and reagent costs for downstream library preparation steps while also minimizing the technical variability among samples. Furthermore, since the 3’Pool-seq protocol uses oligo-dT primers linked to standard indexed TruSeq i7 adaptors (unlike the custom adapter primer sequences used in Drop-seq), the resulting 3′-end cDNA fragments can be easily PCR-amplified using standard TruSeq i7 and Nextera i5 primer reagents. The use of indexed Nextera i5 adapter primers for 3′-end cDNA fragment amplification also enables further barcoding and multiplexing of multiple sample pools into a superpool. The final sequencing library product is a dual indexed hybrid Nextera/Truseq library that maintains strand orientation, with 3′-end cDNA fragments flanked by an indexed Nextera i5 adapter and an indexed TruSeq i7 adapter, and an average length of 550 basepairs. The indices on the Nextera i5 adapter therefore serve as the pool barcode, and indices on the TruSeq i7 adapter serve as the sample barcode within a pool. This early pooling and dual-indexed multiplexing scheme reduces the number of individual sample preparations needed and cuts down the cost and time for library preparation. Furthermore, since 3’Pool-seq uses the 3′-end fragments to quantify transcript abundance, fewer sequencing reads are needed per sample, further reducing the sequencing cost.

### Gene expression quantification using 3’Pool-seq

**Fig. 2:**
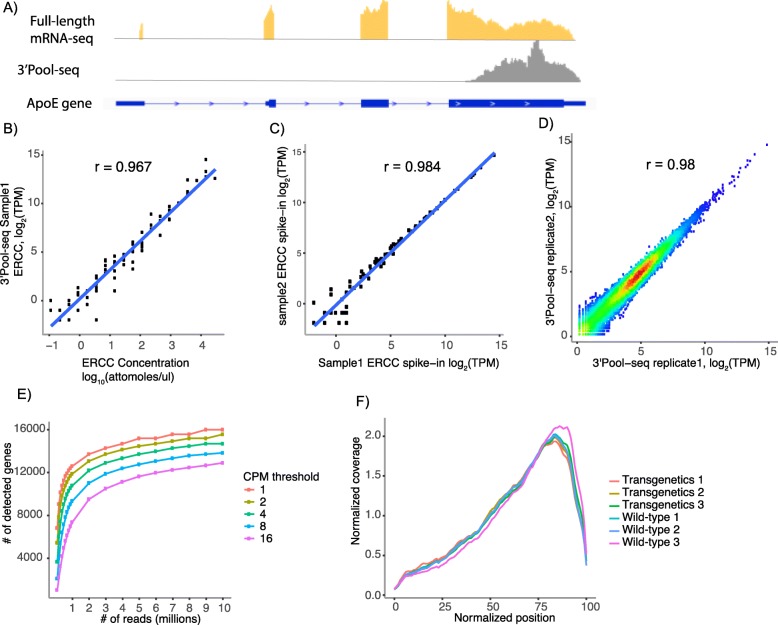
3’Pool-seq provides robust and reproducible gene expression quantification. **a** Read distribution from full-length mRNA-seq (Truseq) and 3’Pool-seq in the *ApoE* gene region. Reads generated using 3’Pool-seq are mapped preferentially towards the 3′-end of the gene. **b** Correlation of the abundance levels of ERCC spike-ins between 3’Pool-seq quantifications and actual pre-mixed concentrations. **c** Correlation of the abundance levels of ERCC spike-ins between 3’Pool-seq replicates. **d** Correlation of gene expression values (log_2_TPM) between 3’Pool-seq replicates. **e** Number of genes detected with different minimal abundance thresholds at increasing read depths (i.e. total number of reads uniquely aligned to gene features). **f** Distribution of 3’Pool-seq reads is skewed towards the 3′-end of the gene body as expected. Normalized positions 0 and 100 correspond to 5′-end and 3′-end of genes, respectively

The performance of 3’Pool-seq was first assessed in terms of its accuracy, sensitivity, and reproducibility in quantifying gene expression. Sequencing libraries were generated using 3’Pool-seq and TruSeq from total RNAs purified from brain cortical samples of three wild-type (WT) C57BL/6 mice and three GFAP-IL6 mice [20]. The GFAP-IL6 mouse is a model that we and others have utilized to study the role of neuroinflammation in neurological and psychiatric disorders [21]. For 3’Pool-seq, on average, 6.4 million 75 base-pair single-end sequencing reads were generated for each sample. Reads were then trimmed for polyA at the 3′-end in case sequencing extended into the polyA tails. After trimming, reads were aligned to the reference genome. Those reads uniquely aligned and mapped to gene feature regions were counted (See Methods for details). A side-by-side comparison of the alignment and gene feature mapping metrics between 3’Pool-seq and TruSeq samples are shown in Table 1. The majority of the 3’Pool-seq reads (87% of total reads) can be mapped to the reference genome, comparable to mapping rates for TruSeq samples (94%). The percentage of uniquely mapped reads for 3’Pool-seq (72%) is slightly lower than Truseq (87%), likely reflecting the higher sequence similarity at the 3′-end of mRNAs. Only 2% of reads were assigned to rRNAs, indicating the oligo-dT primed reverse transcription procedure is efficient in avoiding rRNA contamination. As expected, a higher percentage (42 ± 0.7%) of the 3’Pool-seq reads were mapped to 3′ Untranslated Regions (UTR). As an example, the read distribution in the genomic region around the *Apoe* gene is shown in Fig. 2a. 3’Pool-seq gave a single peak at the last exon of the *Apoe* gene covering the 3’UTR and the 3′-end of the protein coding region while Truseq reads were mapped throughout the gene body. The distribution of reads for the top 1000 most abundant genes is also highly biased towards to 3′-end of the gene body as expected for 3’Pool-seq (Fig. 2f). A more detailed list of sequence counts on a per-sample basis can be found in Additional file 2: Table S1.

**Table 1:** Sequencing and mapping quality metrics comparison between 3’Pool-seq and TrusSeq. Shown in the table are the mean and standard deviation of the different quality metrics

Quality Metrics	3’Pool-seq	mRNA TruSeq
# of samples	6	6
Reads per sample (Millions)	6.4 ± 3.6	33 ± 10.4
Number Uniquely Mapped Reads (Millions)	4.7 ± 2.7	28.7 ± 8.7
% mapped reads	87.2 ± 2	94.4 ± 1.9
% Uniquely mapped reads	72 ± 4	87 ± 1
% coding reads	24 ± 0.8	36 ± 2
% UTR reads	42 ± 0.7	34 ± 0.2
% rRNA reads (× 10^-5)	2 ± 0.4	19.8 ± 8
% non-mRNA reads	31 ± 2	28 ± 3
# of genes detected (TPM >1)	13,571 ± 179	14,135 ± 211
ERCC correlation with theoretical concentrations (r2)	0.93 ± 0.01	0.87 ± 0.03
ERCC pairwise correlation between samples (r2)	0.97 ± 0.01	0.95 ± 0.01

To assess the accuracy of gene expression quantification, an ERCC spike-in mix of 92 synthetic mRNAs with pre-determined concentrations was added to the input total RNA samples prior to library preparation. 3’Pool-seq derived expression values were then compared to theoretical ERCC spike-in concentrations. An average Pearson correlation coefficient r of 0.968 was observed, indicating gene expression quantification from 3’Pool-seq is highly accurate (Table 1). A correlation plot between observed and theoretical ERCC levels in one representative sample is shown in Fig. 2b. An excellent correlation of ERCC quantification between sample replicates (average Pearson’s correlation coefficient r = 0.984, example shown in Fig. 2c) was also observed. It is worth noting that for both ERCC metrics, 3’Pool-seq outperformed TruSeq slightly (Table 1). In addition, a strong correlation between samples was also observed for the expression levels of all genes, as shown in the example in Fig. 2d (Pearson’s correlation coefficient r = 0.98). To assess the sensitivity of 3’Pool-seq at different sequencing depths, we down-sampled reads gradually from 10 million uniquely mapped reads to half a million uniquely mapped reads and assessed how many genes can be detected at different abundance thresholds (Fig. 2e). While the number of genes detected generally decreases as the number of uniquely mapped reads is reduced, the inflection point appears to be at around 1 to 2 million uniquely mapped reads, where the number of genes detected reduces rapidly with continued down-sampling. This suggests that ~ 2 million uniquely mapped reads would be minimally recommended for 3’Pool-seq. These performance metrics, taken together, indicate that 3’Pool-seq is highly accurate, reproducible, and sensitive in gene expression quantification.

### Performance of 3’Pool-seq in detecting differential gene expression

**Fig. 3:**
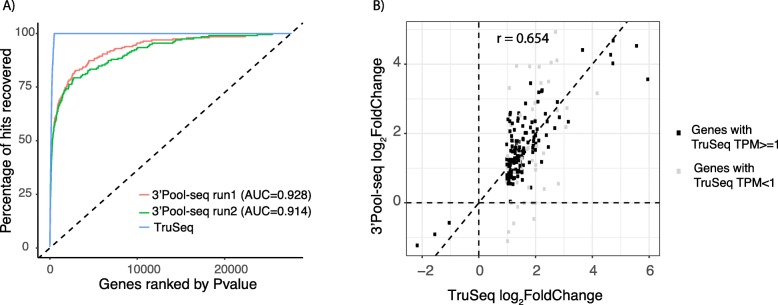
Performance of 3’Pool-seq in detecting differential expressed genes. **a** Differentially expressed genes identified by TruSeq (FDR q-value< 0.05, absolute log_2_(Fold-Change) > 1) were used as the “true DE genes”. **b** Correlation of the log_2_(Fold-Change) quantified by 3’Pool-seq and TruSeq for DE genes identified by the TruSeq protocol

Transcriptional profiling experiments are often designed to study differential expression patterns between conditions ([4, 5] as examples). To assess the ability of 3’Pool-seq to detect differentially expressed genes (DEGs) it was benchmarked against the TruSeq protocol. In total, 194 differentially expressed genes (FDR qvalue< 0.05, absolute log_2_ (Fold-Change) > 1) were identified by TruSeq when comparing GFAP-IL6 transgenic animals to wild-type animals. DEGs are primarily up-regulated genes related to neuroinflammation pathways induced by the expression of pro-inflammatory cytokine IL6. With these DEGs identified from TruSeq, we constructed a Receiver Operating Characteristics (ROC) analysis to assess the recall rate of TruSeq DEGs by 3’Pool-seq where genes were ranked by their differential expression *p*-value. We also conducted two separate 3’Pool-seq library preparations on the same set of samples to assess the technical reproducibility of 3’Pool-seq. Overall, the two technical replicate experiments performed similarly in the ROC analysis with high recall rates for the TruSeq DEGs (average AUC = 0.921, Fig. 3a). In addition, the effect size of the DEGs (i.e. expression fold changes between GFAP-IL6 and wild-type animals) quantified by 3’Pool-seq and TruSeq are correlated with a Pearson’s correlation coefficient r = 0.654 (Fig. 3b).

### Robustness of 3’Pool-seq in low-input samples

**Fig. 4:**
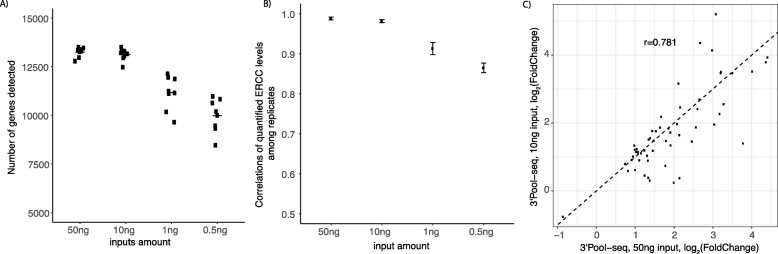
Performance of 3’Pool-seq with low RNA input samples. **a** Number of genes detected (TPM > 1) when different RNA input amounts were used. **b** Correlations of ERCC spike-ins among replicates when different amounts of RNA input were used and ERCC spike-ins were diluted proportionately. **c** Comparisons of log_2_(Fold-Changes) for DE genes (defined as FDR q-value< 0.05, log_2_(Fold-Change) > 1 in the 3’Pool-seq run with 50 ng RNA input) between 10 ng input RNA 3’Pool-seq run and 50 ng input RNA 3’Pool-seq run

Full-length RNA-seq library preparation protocols such as TruSeq often have a minimal requirement of 100-200 ng input total RNA, limiting their utility in studies with scarce sample quantity. Here, the performance of 3’Pool-seq was tested with different input amounts of total RNA, ranging from 0.5 ng to 50 ng. As shown in Fig. 4a, in general more genes can be detected (TPM > 1) as the amount of RNA input increases but the number of genes detected starts to saturate at around 10 ng of RNA input, with a total number of 13,125 genes detected on average. Similarly, stronger gene expression correlations were observed among replicates when higher amounts of RNA inputs were used (Fig. 4b). High global gene expression correlations among replicates (Pearson correlation coefficient r > 0.96) were observed even when as little as 10 ng total RNA inputs were used. In addition, the DEGs detected are comparable between the 10 ng and 50 ng total RNA input runs with their log_2_(Fold-Change) values correlated with a Pearson correlation coefficient *r* = 0.781 (Fig. 4c).

### Plate-based 3’Pool-seq

**Fig. 5:**
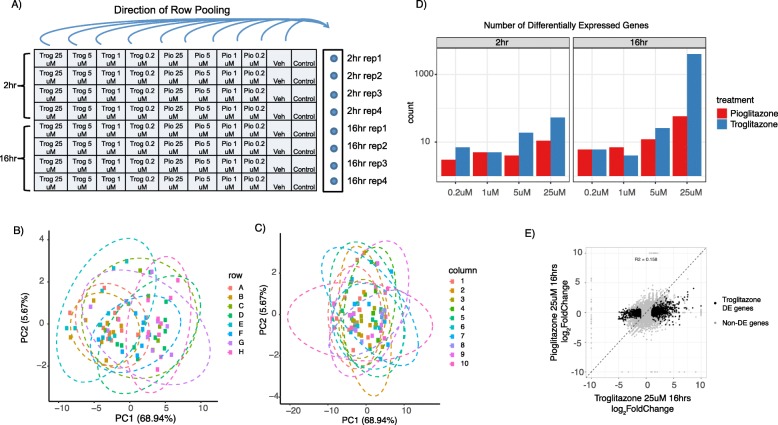
Plate-based format of 3’Pool-seq applied to differentiate gene expression responses between troglitazone and pioglitazone treatments. **a** Layout of plate-based 3’Pool-seq using row pooling scheme. Principal component analysis using ERCC spike-ins is used to assess row effect **b** and column effect **c**. 95% confidence eclipses are shown for each row or column groups. Row effect is observable as indicated by the strong correlation of row groups with PC1 (R^2^ = 0.53), while column effect is not observed (correlation of column groups with PC1 R^2^ = 0.11). **d** Differentially expressed genes identified at different doses and time points for the two PPARγ agonists. Row I.D.s were used in the differential expression analysis to correct for row pooling effect. **e** DE genes identified upon 16 h 25 μM troglitazone treatment showed little differential changes in 16 h 25 μM pioglitazone treatment

The 3’Pool-seq library preparation protocol was further adapted to a 96-well plate format to enable high-throughput RNA-seq profiling experiments. The 96-well format is ideally suited for the 3’Pool-seq dual indexing scheme where samples from either each column or row can be barcoded using the TruSeq i7 indices and pooled after the reverse transcription step. The Nextera i5 indices can then be used as the pool indices. For example, a row pooling scheme would require 12 TruSeq i7 indices (column indices) and 8 Nextera i5 indices (row indices), and the combination of row and column indices can uniquely identify each sample in the 96-well plate format (Fig. 5a). As a test case, we examined the effect of two PPARγ agonist drugs, troglitazone and pioglitazone, in HepG2 cells at multiple doses and time points. Troglitazone is known to have liver cytotoxicity while pioglitazone has a better safety profile [22]. A total of 80 samples were formatted into 8 rows by 10 columns on a 96 well plate and a row pooling scheme was applied as shown in Fig. 5a.

While the row- or column-pooling is convenient and minimizes the within-pool technical variability, it is also important to recognize the potential confounds introduced by pooling. For example, in a row pooling scheme, the different TruSeq i7 indexed primers (column indices) might have slightly different concentrations or efficiencies and render a column-based confounding effect. Similarly, experimental variabilities introduced after the row pooling would affect all samples in the same pool and appear as row-based confounding effects. While certain confounding effects can be minimized, for example, by carefully selecting high-quality primers and equalizing primer concentrations, other confounding effects such as those introduced after pooling are harder to avoid. Therefore, additional procedures were incorporated in our experimental and computational analysis workflow to quantify and correct for these potential row- and column-based confounding effects. Equal amounts of ERCC standards were spiked in to all input RNA samples. After library preparation and sequencing, we quantified the ERCC concentrations from sequencing reads, and computationally assessed potential column and row effects through principal component analysis (PCA). Once observed, these column or row effects could be incorporated into the differential gene expression analysis as a covariate to improve DEG calls. Figure 5b shows the PCA analysis of the ERCC spike-ins quantified in our PPARγ test case. The samples from different pools separate clearly along the first principal component (coefficient of determination of rows with PC1 R^2^ = 0.53), indicating a strong row effect. In contrast, no obvious column effect was observed (coefficient of determination of columns with PC1, R^2^ = 0.11, Fig. 5c). After incorporating the row effect into the differential expression analysis as a covariate, a total of 2172 DEGs (absolute log2(Fold-Change) > 1 and FDR q-value< 0.05) were observed at the highest dose (25 μM) 16-h treatment of troglitazone, while only <70 DEGs were found in similar pioglitazone treatments. GO enrichment analysis further confirmed that genes annotated with “regulation of cell death” (GO:0010941) were highly enriched among the DEGs triggered by troglitazone (enrichment *p*-value< 8.7E-15), consistent with the previously reported cytotoxicity [22]. Interestingly, many of these DEGs show little gene expression changes at lower doses and earlier time points (Fig. 5d), illustrating the importance of testing compounds at multiple doses and time points. It is also worth noting that, without correcting the row pooling effect in the differential expression analysis, fewer significantly differentially expressed genes (1707 DEGs) could be identified, further emphasizing the need to utilize ERCCs to assess column and row effects and incorporate them into differential gene expression analysis.

## Conclusions

Gene expression is a highly dynamic process. The effect of genetic regulation or external perturbations on gene expression is highly time- and dose-dependent. While whole transcriptome profiling is a powerful technique that enables genome-wide interrogation of gene expression, current practices are often limited to taking snapshots of the transcriptome at a single condition due to the cost and time required for traditional RNA-seq experiments. Thus, the 3’Pool-seq method presented here provides a cost- and time-effective solution for large-scale RNA-seq studies, enabling thorough interrogation of transcriptome changes at multiple time points and conditions.

The 3’Pool-seq method integrates several technology advancements, leveraging the 3′-barcoding and early pooling strategies commonly used in single-cell RNA-seq studies and template switching and tagmentation techniques for efficient cDNA amplification and fragmentation. The reduced and optimized reaction volumes further save on library preparation reagents. By using standard TruSeq i7 and Nextera i5 indexed primers, the final 3’Pool-seq libraries are fully compatible with standard Illumina sequencing protocols without the need for any custom sequencing reagents. Overall, the 3’Pool-seq library preparation method costs ~$3 per sample and requires only 2–3 h hands-on time (Table 2), significantly reducing the cost and time for library preparation. Furthermore, it was demonstrated that 3’Pool-seq generated high quality libraries with > 80% of reads mappable to the reference genome and a majority (> 66 ± 1.5%) of the uniquely mapped reads located in usable gene feature regions, as well as a very low percentage of reads (< 1.9 ± 0.8%) from rRNA and genomic DNA contamination (Table 1). By using ERCC spike-in standards, it was shown that the 3’Pool-seq method was able to accurately and reproducibly quantify gene expression levels. More importantly, 3’Pool-seq was able to reproduce the differentially expressed genes from the standard TruSeq protocol at a small fraction (5%) of the library preparation cost and one third of the hands-on time. Our down-sampling procedure showed minimally 2 million gene feature aligned reads (~ 4 million raw reads) would capture the majority of the expressed genes, allowing efficient multiplexing of a large number of samples in a single sequencing run.

**Table 2:** Cost, Time, and Qualitative Metrics comparison of 3’Pool-seq and TrusSeq, as well as two additional 3′-end sequencing techniques: Plate-Seq and DRUG-seq. (N/A) indicates that values were not readily accessible in the corresponding article. (*) Represents sequencing costs on a HighSeq platform, while others represent costs on a NextSeq platform

	3’Pool-seq	TruSeq	Plate-Seq	DRUG-seq
Library prep cost per sample	$3	$60	$3	$0.2–1
Sequencing cost per sample	$12	$100	$12*	$2–4*
Overall time for library prep	8–12 h	2–3 days	> 2 days	N/A
Hands-on time	2–3 h	6–8 h	N/A	N/A
Samples per Run	96	12–24	96	384–1536
Major Advantage	No custom equipment or sequencing primers, stringently benchmarked against ERCC and TruSeq.	Best option for detecting low-abundance genes or splice variants.	Oligo-dT Plate-based RNA purification.	Most affordable option, highest throughput, manual alternative is described.
Major Disadvantage	Involves RNA purification step, lowest throughput of three 3′-end techniques described herein.	Most expensive option, low-throughput, technically tedious.	Requires custom liquid dispensing equipment, no detailed benchmarking with ERCC or TruSeq.	Requires custom liquid dispensing equipment, manual protocol not benchmarked.

In accordance with research that compared commercial 3′-end sequencing with full-length RNA-seq [23], we found that full-length sequencing with TruSeq did in fact detect more differentially expressed genes than 3’Pool-seq (Additional file 1: Figure S1.A). A deeper analysis reveals that the differentially expressed gene set which is unique to TruSeq has a longer average length than the other sets and is on average expressed at a relatively lower level (Additional file 1: Figure S1.B). This is likely explained by the observation that full-length RNA-seq has a bias towards detecting longer transcripts due to the fact that they contribute more fragments per sequencing run [24]. Not surprisingly, the lengths of the differentially expressed genes detected by 3’Pool-seq do not show a size bias (Additional file 1: Figure S1.B). While previous research has shown a higher correlation of DEG magnitude between 3′-end sequencing and full-length sequencing [23], different methods were used (Lexogen QuantSeq and Kapa Stranded mRNA-Seq, respectively). Further studies would therefore be required to determine how 3’Pool-seq and/or TrueSeq compare to these methods. Additional studies using, for example, different sets of ERCC standards at different concentrations will also be required to simulate differential gene expression in a system with a known ground-state to truly evaluate the false positive and negative hit rate of each method. Regardless, a gene ontology (GO) analysis of the DEGs uncovered by 3’Pool-seq and TruSeq reveals almost identical pathways (Additional file 3: Tables S2 and Additional file 4: Table S3, respectively), further supporting the validity of using 3′-end sequencing to study transcriptomic responses to system perturbations.

Another innovation of the 3’Pool-seq method is the support for 96-well plate format for library preparation through row or column-based pooling, and the use of ERCC spike-ins and computational procedures to assess and correct for pooling confounding effects. As shown in the PPARγ test experiment, proper design of the pooling strategy and the correction of row or column-based pooling confounds are critical for differential gene expression analysis. Furthermore, the 96-well plate based 3’Pool-seq library preparation format can easily be adapted for automation.

Several low-cost RNA-seq library preparation techniques have been reported recently, each with their strengths and weaknesses (18,19). To evaluate the performance of any given methodology, it is important to consider its robustness in several different areas. These include ERCC measurement accuracy, DEG detection as compared to TruSeq, throughput, general accessibility, and sequencing metrics such as mapping rate. Without a true head-to-head comparison of different techniques using identical samples, their relative strengths and weaknesses can only be determined by evaluating their performances with the above criteria. Both PLATE-Seq and DRUG-seq were shown to have throughput capabilities that are on par with L1000 [18, 19], while still being able to directly detect the full transcriptome. However, this comes at the cost of using custom sequencing primers, sophisticated equipment, and, in the case of PLATE-Seq, specialized oligo-dT purification plates. Furthermore, these papers do not report performance metrics such as ERCC measurements. An ROC analysis comparing DRUG-seq to TruSeq was performed and it gave an average AUC of 0.73 (19), as compared to the 0.921 value generated in the 3’Pool-seq experiments. The DRUG-seq paper also describes a manual alternative of their protocol that does not require liquid-handling equipment, but it does not discuss its performance in detail [19]. These differences are summarized in Table 2. In this paper we have described the strengths of 3’Pool-seq with regard to accurate ERCC measurements, quality metrics such as mapping rate, and DEG detection that is on par with TruSeq.

With much reduced cost, streamlined experimental procedures, high data quality for gene expression quantification and differential analysis, robust performance with low RNA inputs, and flexible support for plate-based library format, 3’Pool-seq not only provides significant cost and time saving for existing RNA-seq applications but also opens up new opportunities for future large-scale transcriptomics studies.

## Methods

### Animal care and dissection

All procedures were performed in compliance with the National Institutes of Health Guide for the Care and Use of Laboratory Animals under the approval of the Pfizer Cambridge Institutional Animal Care and Use Committee. 3-month-old GFAP-IL6 homozygous or wild-type mice were euthanized by cervical dislocation followed by decapitation. Frontal cortex was dissected and snap-frozen in RNAse-free tubes on dry ice. Total RNA was isolated by Trizol-chloroform extraction.

### HepG2 culture, treatment, and crude RNA preparation

HepG2 cells (ATCC) were cultured in growth media (DMEM supplemented with 1% Penn/Strep, 1% MEM, 1% Glutamax, and 10% Serum) at 37 °C, 5% CO_2_ and 85% relative humidity. For treatments, 10^5^ cells in 1 ml growth media were seeded into 24-well culture dishes and allowed to settle for 6 h. Growth media was then replaced with serum-free media containing the indicated concentrations of troglitazone or pioglitazone (Sigma-Aldrich) and vehicle (DMSO, 0.1% final concentration), and the treated cells were allowed to grow for 2 or 16 h. Cells were then stripped of media, washed once with PBS, and were lysed by the addition of 1 ml Trizol. Total RNA was then isolated by Trizol-chloroform extraction.

### RNA refinement and quantification

RNA from Mouse Brains or HepG2 cells was further refined with an RNeasy Micro kit (Qiagen) using the standard RNA Cleanup protocol, starting with 100 μl of crude RNA obtained above. Refined RNA was then examined with an Agilent TapeStation 4200 (Agilent Technologies, Inc) to ensure that all samples had a RIN value greater than 8.0 and was quantified with a Qubit 3.0 Fluorometer (Life Technologies).

### Reverse transcription, pooling, and exonuclease treatment

All oligo-nucleotides used in this study were sourced from Integrated DNA Technologies, Inc. as PAGE-pure oligos, and the sequences can be found in the Additional file 5 Primer Sequences section.

The indicated amount of RNA was diluted in 5 μl RNAse-free water and plated in 96-well plates. 1 μl Indexed RT Primer (10 μM), 1 μl 10 mM dNTP Mix (New England Biolabs), and 1 μl diluted ERCC Spike-In Mix 1 (0.004 μL stock ERCC per μg RNA, ThermoFisher) was added to RNA. Annealing was initiated by placing the plate in a thermocycler at 72 °C for 3 min, followed by immediate placement on ice.

Next, 10 μl of a Master Mix containing 3.6 μl SuperScript 5x Buffer, 0.25 μl H_2_O, 0.25 μl DTT (100 mM), 2 μl Betaine (5 M), 0.9 μl MgCl_2_ (100 mM), 2.5 μl Template Switching Oligo (10 μM) and 0.5 μl SuperScript II Reverse Transcriptase (ThermoFisher) was added to each sample, and Reverse Transcription was carried out in a thermocycler using the following program: 42 °C for 90 min, 10 cycles of (50 °C for 2 min, 42 °C for 2 min), 70 °C for 15 min, and 4 °C hold.

Samples were pooled by mixing an equal volume of each Reverse Transcription reaction into a new well at a total volume of 20 μl. Residual primers were then degraded with the addition of 1 μl Exonuclease I (New England Biolabs) and incubated at 37 °C for 45 min followed by denaturation at 92 °C for 15 min.

Reverse transcription reactions were then cleaned by adding 12 μl of Agencourt XP Beads (Beckman Coulter) to each pool of samples. Manufacturer’s suggestions were followed, and cDNA was eluted with 10 μl Elution Buffer and transferred to a new well.

### cDNA amplification and Tagmentation

To each cDNA pool we added 1.25 μl Enrichment Primer A (20 μM), 1.25 μl Enrichment Primer B (20 μM), and 12.5 μl Kapa HiFi HotStart Ready Mix (Kapa Biosystems). Amplification was then carried out in a thermocycler for the following Touch-Up PCR program: 95 °C for 3 min, 4 cycles of (98 °C for 20 s, 65 °C for 45 s, 72 °C for 3 min), 9 cycles of (98 °C for 20 s, 67 °C for 20 s, 72 °C for 3 min), 72 °C for 5 min, and a 4 °C hold.

PCR reactions were then cleaned by adding 15 μl of Agencourt XP Beads (Beckman Coulter) to each well and mixing. Manufacturer’s suggestions were followed, and cDNA was eluted with 10 μl Elution Buffer and transferred to a new well. Amplified cDNA was then quantified with a Qubit 3.0 Fluorometer (Life Technologies).

Pools of amplified cDNA were then subjected to Tagmentation via Nextera XT DNA Library Preparation (Illumina, Inc.) according to the manufacturer’s protocol with the following modifications: cDNA is diluted to 0.4 ng/μl. Next, we used a Tagmentation time of 3 min as opposed to the recommended 5 min. We also used our own Indexed Nextera i5 Primers (2 μM) and Enrichment Primer A (2 μM) in the PCR step. Lastly, all the volumes were cut down by 5-fold universally in order to maximize the number of reactions per kit.

### NGS library evaluation and loading

NGS library pools that were generated from the Nextera XT procedure were examined qualitatively in an Agilent TapeStation 4200 (Agilent Technologies) to determine average library lengths, and quantitatively in a Qubit 3.0 Fluorometer (Life Technologies). NGS library molarity was then calculated using 660 g/mol per base-pair as a molecular weight.

NGS libraries were then diluted to 4 nM, mixed in equal volumes to create a superpool, and prepared for sequencing in the NextSeq 500 (Illumina) according to manufacturer’s suggestions. Single-end sequencing reactions were performed with a 75-cycle High V2 kit (Illumina) and the following settings: Read 1: 70 bases, Index 1: 6 bases, Index 2: 8 bases.

### Bioinformatic analysis

3’Pool-seq data can be processed with standard RNA-seq pipelines with simple modifications. After standard sample de-multiplexing (bcl2fastq), an extra step was added to trim off polyA sequences (minimal length of 12 nucleotides) located towards the 3′-end of the reads (after 25th position), as sequencing reads from shorter fragments could extend into the polyA tails of mRNA transcripts. We found this trimming step often improves the alignment rate of reads. Trimmed reads were aligned to a reference genome (mm10 for mouse and hg19 for human) using STAR aligner (version 2.4, [25]) with the following parameters (−-alignSJDBoverhangMin 1 --outFilterMismatchNoverLmax 0.1 --alignIntronMax 1,000,000). The same STAR aligner parameters were also used for aligning reads from TruSeq samples. Reads aligned to annotated gene features (GENCODE vM6 for mouse and GENCODE v19 for human) were counted using featureCounts (version 1.6.3, [26]).

Mapping quality metrics were collected using PICARD (https://broadinstitute.github.io/picard/). Since 3’Pool-seq sequences only the 3′-end of mRNA transcripts, no gene length normalization was applied to read counts when calculating Transcripts Per Million (TPM) values. Differential gene expression analysis was carried out using the DESeq2 package in R [27]. For the plate-based 3’Pool-seq study of troglitazone and pioglitazone treated samples, row number (i.e. pool id) was included in the DESeq2 differential analysis as a categorical covariate to adjust for the observed row effect.

Principal component analysis, ROC analysis, and other custom statistical analyses were carried out using R software (version 3.1). Gene Ontology functional enrichment analysis of troglitazone induced gene expression changes were performed using Panther [28].

## Supplementary information

**Additional file 1: Figure S1.** A Comparison of DEGs detected by TruSeq and 3’Pool-Seq. A) Venn Diagram depicting the DEGs that are detected by TruSeq, and/or 3’Pool-Seq at the indicated cutoffs. B) A histogram showing Mean TPM, transcript length, and absolute log_2_(Fold-Change) distributions of DEGs detected by TruSeq and/or 3’Pool-seq.

**Additional file 2: Table S1.** A per-sample overview of sequencing metric details that were used to construct Table 1 of the main manuscript.

**Additional file 3: Table S2.** A Gene Ontology analysis of the pathways ranked by *p*-value represented by the DEGs detected by 3’Pool-seq in the Wild-Type vs. GFAP-IL6 mouse model.

**Additional file 4: Table S3.** A Gene Ontology analysis of the pathways ranked by p-value represented by the DEGs detected by TruSeq in the Wild-Type vs. GFAP-IL6 mouse model.

**Additional file 5.** Supplemental Material Primer Sequences. A list of all oligo-nucleotides employed in this study, using sequence conventions as outlined by IDT, Inc.

## Data Availability

Transcriptional fastq files have been deposited in the GEO repository under accession number GSE125571. https://www.ncbi.nlm.nih.gov/geo/query/acc.cgi?acc=GSE125571

## Abbreviations

WT: Wild-Type
DEGs: Differentially Expressed Genes
GO: Gene Ontology
NGS: Next Generation Sequencing
RNA-seq: RNA sequencing
ROC: Receiver Operating Characteristics
TPM: Transcripts Per Million
UTR: Untranslated Regions
