# Supplementary material for: 3’Pool-seq: an optimized cost-efficient and scalable method of whole-transcriptome gene expression profiling
Source: BMC Genomics. 2020 Jan 20;21:64. doi: 10.1186/s12864-020-6478-3 (PMC6971924; doi:10.1186/s12864-020-6478-3)
Supplement: Supplementary file 1 — Additional file 1: Figure S1. A Comparison of DEGs detected by TruSeq and 3’Pool-Seq. A) Venn Diagram depicting the DEGs that are detected by TruSeq, and/or 3’Pool-Seq at the indicated cutoffs. B) A histogram showing Mean TPM, transcript length, and absolute log2(Fold-Change) distributions of DEGs detected by TruSeq and/or 3’Pool-seq. [file 12864_2020_6478_MOESM1_ESM.pptx]

## Slide 1
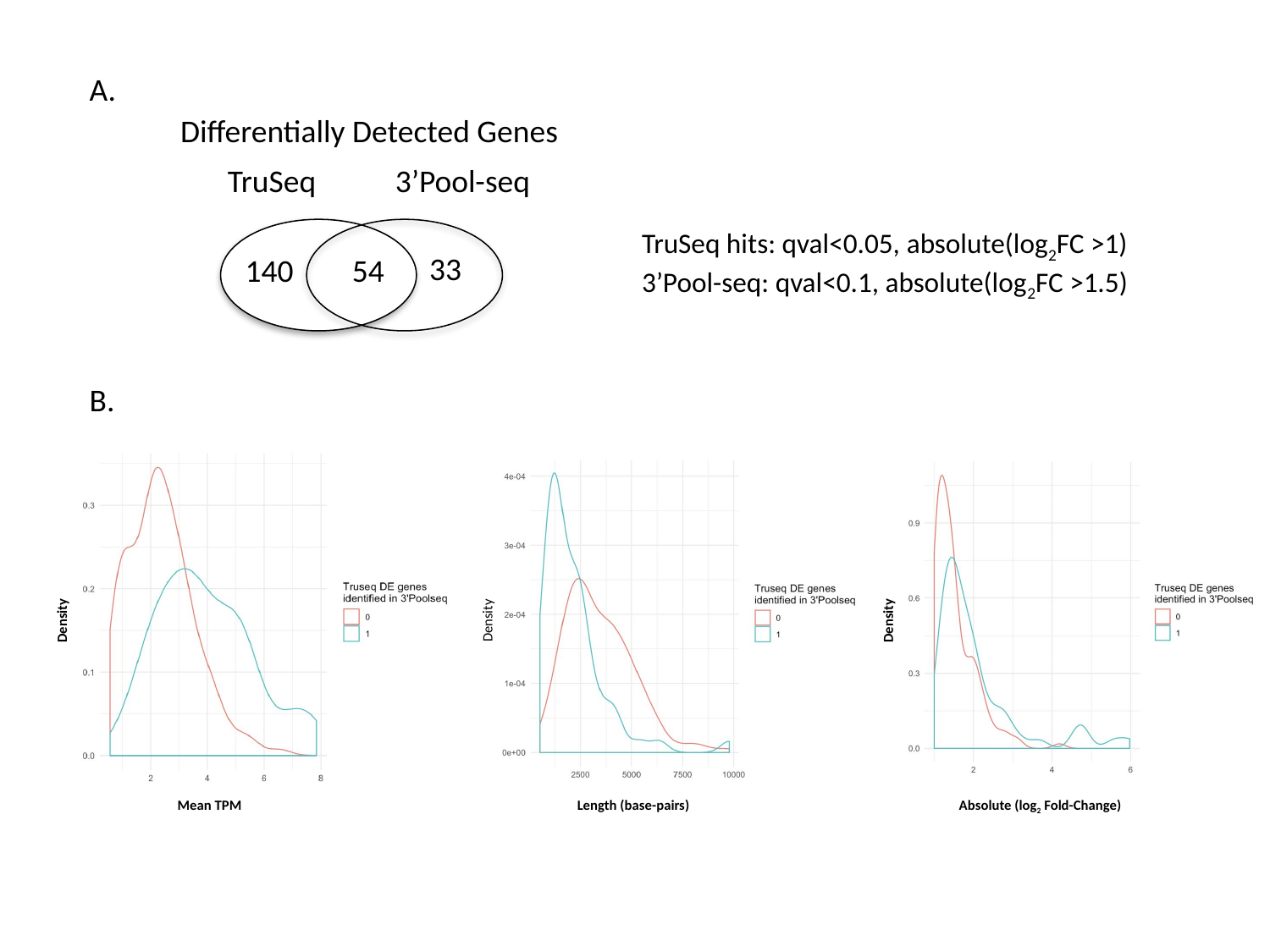

A.
Differentially Detected Genes
TruSeq
3’Pool-seq
TruSeq hits: qval<0.05, absolute(log2FC >1)
3’Pool-seq: qval<0.1, absolute(log2FC >1.5)
33
54
140
B.
Density
Density
Density
Mean TPM
Length (base-pairs)
Absolute (log2 Fold-Change)
